# Simultaneous RP-HPLC Estimation of Trifluoperazine Hydrochloride and Chlordiazepoxide in Tablet Dosage Forms

**DOI:** 10.4103/0250-474X.58192

**Published:** 2009

**Authors:** Sejal K. Patel, N. J. Patel

**Affiliations:** S. K. Patel College of Pharmaceutical Education and Research, Ganpat University, Kherva, Mehsana-382 711, India

**Keywords:** Trifluoperazine HCl, chlordiazepoxide, RP-HPLC

## Abstract

A binary mixture of trifluoperazine HCl and chlordiazepoxide was determined using reversed-phase liquid chromatography method using methanol:water (97:03, v/v) pumped at a flow rate of 1.0 ml/min. Quantification was achieved with ultraviolet detection at 262 nm over concentration ranges of 0.1-1 and 0.5-5 μg/ml; mean accuracies were 101.05±0.47 and 98.97±0.33 %, respectively. The method was successively applied to tablet dosage forms as no chromatographic interferences from the tablet excipients were observed. The method retained its accuracy and precision when the standard addition technique was applied.

Trifluoperazine HCl (TFP) is official in IP, BP and USP. Both IP[1] and USP[2] describe non-aqueous titration method whereas BP[3] describes UV spectroscopy method for estimation of TFP. A literature survey revealed spectrophotometric[4,5], HPLC[6], HPTLC[7] methods for simultaneous estimation of TFP in pharmaceutical formulation with other drugs. Chlordiazepoxide (CLR) is official in IP, BP and USP. The IP[8], BP[9] and USP[2] describe non-aqueous titration, potentiometry titration and HPLC methods, respectively for estimation of CLR. Literature survey indicated difference spectrophotometric[10], micellar liquid chromatography[11], derivative spectrophotometry[12] methods for CLR with other drugs in pharmaceutical formulations. Literature survey revealed spectrophotometry method[13] and RP-HPLC method[14] for the simultaneous determination of these two drugs. The present RP-HPLC method uses simple mobile phase ratio (methanol: water- 97:03), higher sensitivity and analysis will complete before 6 min. Therefore the present study was to determine both drugs concurrently by sensitive, accurate, rapid and precise RP-HPLC method for routine analysis.

The chromatography was performed on a Shimadzu (Columbia, MD) RP-HPLC instrument (LC-2010CHT) equipped with PDA detector, Phenomenex (Torrance, CA) C18 column (250×4.6 mm id, 5 μm particle size) was used as stationary phase. Standard samples of TFP and CLR and market samples of Serepose tablets (Unimarck Pharma, Chandigarh), each tablet contained 1 mg TFP and 10 mg CLR were used. Triple distilled water, methanol (S. D. Fine Chemical, Ahmedabad, India) used were of HPLC grade.

TFP and CLR stock solutions (40 μg/ml and 200 μg/ml, respectively) were prepared by weighing accurately 2 mg TFP and 10 mg CLR powder into 2 separate 50 ml volumetric flasks; 25 ml methanol was added, shaken for a few minutes, and diluted to volume with methanol. From these solutions (2.5 ml) were transferred into 2 separate 10 ml volumetric flasks and diluted to the mark with methanol to give final concentrations of 10 and 50 μg/ml, respectively. Accurate aliquots equivalent to 0.1-1 μg TFP from its working solution (10 μg/ml) and aliquots equivalent to 0.5-5 μg CLR from its working solution (50 μg/ml) were transferred into 2 separate sets of 5 ml volumetric flasks and diluted to volume with methanol. Powder from the mixed contents of 20 tablets, equivalent to 1 mg TFP and 10 mg CLR, was transferred accurately to a 50 ml volumetric flask and diluted to volume with methanol. The solution was diluted to the same concentrations of working standard solutions and treated according to the linearity for the RP-HPLC method. The separation was done on a C18 column using methanol:water (97:03, v/v) as the mobile phase. The chromatogram was recorded under the following instrumental parameters: 20 μl injection volume, flow rate, 1.0 ml/min at 40° temperature and the eluent monitored at 262 nm. Calibration curves for both TFP and CLR were plotted, and the corresponding regression equations were calculated.

The aim of this work was to develop sensitive, accurate, precise and rapid analytical method for the simultaneous determination of TFP and CLR. This was achieved using RP-HPLC method. To optimize the proposed RP-HPLC method, all of the experimental conditions were investigated. For the choice of the stationary phase, reversed-phase separation was preferred due to the drawbacks of the normal phase, e.g., hydration of silica with water that can cause peak tailing. To optimize the mobile phase, different systems were tried for chromatographic separation of the two components by combining homogenous design and solvent polarity optimization. The best resolution was achieved using a mobile phase consisting of methanol:water (97:03, v/v), which gave good resolution and sensitivity of both drugs (fig. 1).

**Fig. 1:**
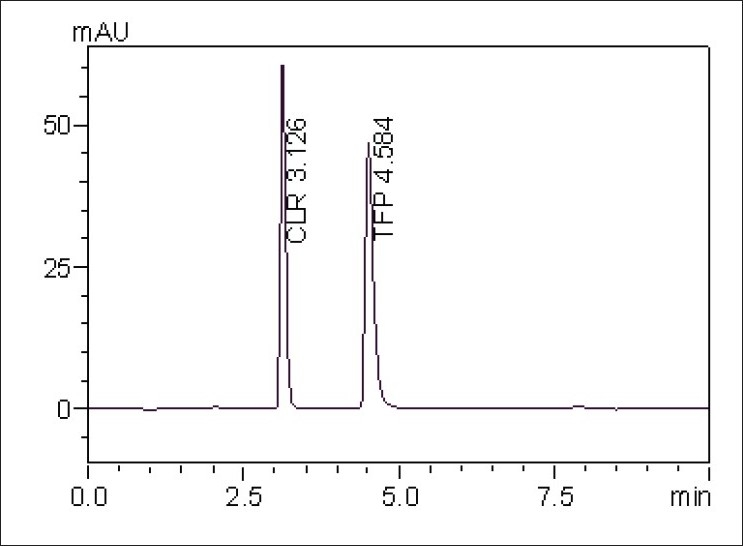
RP-HPLC chromatogram of TFP and CLR at 262 nm TFP is trifluoperazine HCl and CLR is chlordiazepoxide

System suitability testing of the RP-HPLC method gave good relative retention time = 1.46; theoretical plates= 5253.8 and 5283.98; asymmetry factor (A)= 1.12 and 1.30; and tailing factor (T)= 1.12 and 1.34 for TFP and CLR, respectively (Table 1). A linear relation was obtained between peak area and the concentration of the two drugs in the range of 0.1-1 and 0.5-5 μg/ml for TFP and CLR, respectively. The linear regression equations were computed, Y=117926 X+10901, r= 0.9976 and Y=123994 X+35915, r= 0.9979, where Y is the area under the peak, X is the concentration in μg/ml, and r is the correlation coefficient. Results obtained by applying the RP-HPLC procedure showed that TFP and CLR can be simultaneously analyzed in the prepared mixtures with mean recoveries of 101.05±0.47 and 98.97±0.33 %, respectively. The proposed method has been applied to assay TFP and CLR in tablets without any interference from the additives (Table 2). The limit of detection for TFP and CLR were found to be 0.05 μg/ml and 0.05 μg/ml, respectively; the limit of quantification for TFP and CLR were found to be 0.1 μg/ml and 0.5 μg/ml, respectively by visual method. The low % CV values of intra-day (0.35-1.56 for TFP and 0.48-1.31 for CLR) and inter-day (0.48-1.67 for TFP and 0.57-1.62 for CLR) precision reveal that the proposed method is precise. Thus, the proposed procedure can be used in routine analysis.

**TABLE 1:** SYSTEM SUITABILITY TEST PARAMETERS FOR TFP AND CLR FOR PROPOSED METHOD

Parameters	RP-HPLC method	
	
	TFP ± % RSD^a^	CLR ± % RSD^a^
Retention time, min	4.58±0.19	3.12±0.20
Tailing factor	1.12±0.67	1.34±0.56
Asymmetry factor	1.12±0.93	1.30±0.39
Theoretical plates	5253.8±1.13	5283.98±1.33
Repeatability of measurement (n^b^ = 6)	0.67	0.48

a RSD is relative standard deviation

b n is number of determinations, TFP is trifluoperazine HCl and CLR is chlordiazepoxide

**TABLE 2:** ASSAY RESULTS FOR TABLETS USING THE PROPOSED METHOD

Formulation	Amount of drug taken (mg)		Amount of drug found (mg)		% Amount found (n^a^=3) ± SD^b^	
			
	TFP	CLR	TFP	CLR	TFP	CLR
Tablets	1	10	1.00894	9.91332	100.89 ± 0.95	99.13 ± 0.87
	1	10	1.0106	9.8964	101.06 ± 0.20	98.96 ± 1.00

a n is number of determinations

b SD is standard deviation
